# Gallstone in the gastric remnant after bariatric surgery

**DOI:** 10.1093/jscr/rjae524

**Published:** 2024-08-28

**Authors:** Sofia Teixeira da Cunha, Georgios Vergos, Urs Pfefferkorn

**Affiliations:** Clinic for Visceral, Vascular and Thoracic Surgery, Cantonal Hospital Olten, 4600 Olten, Switzerland; Clinic for Visceral, Vascular and Thoracic Surgery, Cantonal Hospital Olten, 4600 Olten, Switzerland; Clinic for Visceral, Vascular and Thoracic Surgery, Cantonal Hospital Olten, 4600 Olten, Switzerland

**Keywords:** bariatric surgery, laparoscopic Roux-en-Y gastric bypass, cholesterol stone, obesity

## Abstract

In the last decades, bariatric surgery has been widely performed to treat obesity and its co-morbidities, with the laparoscopic Roux-en-Y gastric bypass (LRYGB) being the second most commonly performed procedure. Abdominal pain after LRYGB is a common symptom. This report concerns a case of a rare cause of chronic abdominal pain after LRYGB and cholecystectomy in a 48-year-old woman due to a cholesterol stone within the gastric remnant that was removed via open gastrectomy. This is the first documented case of cholesterol stone formation in the gastric remnant and underscores the importance of vigilance for atypical complications in patients undergoing bariatric procedures.

## Introduction

Obesity is one of the most prevalent chronic diseases worldwide. The rise in the incidence of obesity has been associated with the increase in non-communicable diseases (NCD) such as diabetes mellitus, cardiovascular disease, and cancer. Approximately 5 million deaths per year caused by NCDs are related to overweight and obesity [1]. In the last decades, bariatric surgery has been widely performed to treat obesity and its co-morbidities, with the laparoscopic Roux-en-Y gastric bypass (LRYGB) being the second most commonly performed procedure worldwide [2]. Abdominal pain after LRYGB is a common symptom. Different cohort studies show a prevalence between 23% and 43% [3–5]. The causes are heterogeneous, with the most prevalent being related to cholelithiasis and intestinal obstruction, more specifically internal herniation through mesenteric defects.

In some patients, the aetiology of abdominal pain remains unknown. In a retrospective study, Gormsen *et al.* reported a prevalence of 21% of postoperative chronic abdominal pain in a cohort of 787 patients who underwent LRYGB. Preoperative use of strong analgesics, postoperative complications, smoking, and unemployment were documented risk factors for postoperative chronic abdominal pain [4].

The present report addresses a case of a rare cause of chronic abdominal pain after LRYGB.

## Case report

A 48-year-old female presented with chronic postprandial pain in the left upper quadrant for several months. Her past medical history included several abdominal surgeries. Initially, she underwent an LRYGB and cholecystectomy with dorsal cruroraphy with hiatoplasty for a hiatal hernia in 2007, with a body mass index (BMI) of 42.8 kg/m^2^. The patient underwent laparoscopic revision with mesh augmentation due to hiatal hernia recurrence in 2008. Due to mesh erosion into the gastric pouch, the patient underwent mesh explantation including pouch excision and reconstruction with jejuno-oesophagostomy in 2014.

In 2022, the patient presented with colicky left upper quadrant abdominal pain after eating and occasional vomiting. The weight was well controlled with a BMI of 23.6 kg/m^2^.

**Figure 1:**
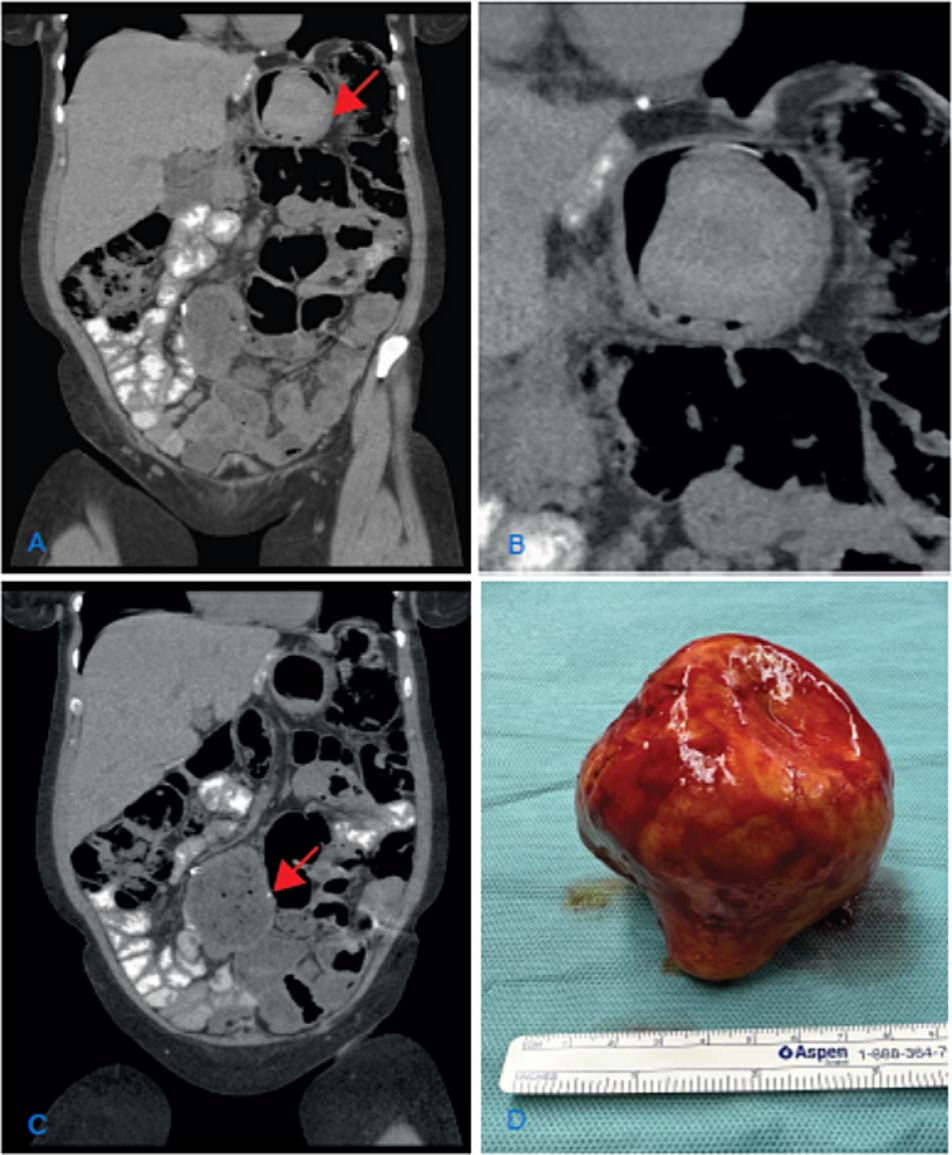
(A) Coronal CT scan image showing the dilatation of the gastric remnant with hypodensemass (arrow); (B) zoom of the gastric remnant with a 5-cm hypodense mass; (C) coronal CT scan image of the dilated JJ-anastomosis (arrow); (D) retrieved specimen a gallstone with approximately 10-cm diameter.

The clinical examination and lab results were normal. A gastroscopy did not reveal any pathological findings. The abdominal CT scan showed dilation of the jejuno-jejunostomy (JJ), biliopancreatic limb, and gastric remnant (Fig. 1A and C). Within the gastric remnant, hypodense mass of at least 5-cm diameter was detected (Fig. 1B). Considering the imaging findings, the patient was proposed for revisional surgery.

An elective laparotomy was performed. By palpation of the gastric remnant, a hard mass was detected. The specimen was retrieved through a gastrotomy. A hard yellow-brown stone ~10 cm in diameter was found intraluminally. After retrieving the intraluminal mass, the dilated side-to-side JJ-anastomosis was resected and reconstructed in an end-to-side fashion.

The chemical analysis confirmed the macroscopic suspicion of a cholesterol gallstone (Fig. 1D). The follow-up was uneventful, and the symptoms resolved.

## Discussion

The diagnosis and treatment of chronic abdominal pain after LRYGB are challenging.

Complications related to the gastric remnant after LRYGB are especially difficult to diagnose and the knowledge of gastric remnant physiology and function remains limited [6]. A case series of 19 patients who underwent LRYGB with postoperative chronic abdominal pain demonstrated on HIDA, the presence of biliary reflux in the gastric remnant and histological signs of chronic gastric inflammation. All patients were treated with remnant gastrectomy, with 16 reporting total remission of symptoms at a mean follow-up of 6.5 years [7]. Some functional studies with hepatobiliary scintigraphy (HIDA) have demonstrated duodenogastric reflux postcholecystectomy. Gastroparesis of the gastric remnant in patients with LRYGB was also described with HIDA [8, 9]. A randomized controlled trial by Tog *et al.* with 126 patients who underwent hiatal hernia repair with mesh versus no-mesh identified revision surgery as an independent risk factor for delayed gastric emptying [10].

Our patient underwent a cholecystectomy at the time of the LRYGB, which might have facilitated bile reflux. The previous hiatal hernia repair and later revision most likely led to gastroparesis by impairing the vagal nerve function and dilated jejunojejunostomy impaired emptying of the biliopancreatic limb [11].

## Conclusion

This is the first documented case of cholesterol stone formation in the gastric remnant. The pathophysiology is most likely multifactorial:

The functional obstruction of the dilated JJ-anastomosis, the previous cholecystectomy, and the impaired gastric emptying due to vagal nerve dysfuntion after previous hiatal repair probably all contributed to the biliary stasis that ultimately caused the gall stone formation in the remnant stomach.

This case nicely illustrates the pathophysiology after LRYGB and underscores the importance of vigilance for atypical complications in patients undergoing bariatric procedures.

## References

[1] Okunogbe A , Nugent R, Spencer G, et al. Economic impacts of overweight and obesity: current and future estimates for 161 countries. BMJ Glob Health 2022;7:e009773. https://doi.org/10.1136/ bmjgh-2022-009773. 10.1136/bmjgh-2022-009773 PMC9494015 36130777

[2] IFSO 8th global register report. https://www.ifso.com/pdf/8th-ifso-registry-report-2023.pdf.

[3] Chahal-Kummen M , Blom-Høgestøl IK, Eribe I, et al. Abdominal pain an symptoms before and after roux-en-Y gastric bypass. BJS Open 2019;3:317–26. 10.1002/bjs5.50148. 31183448 PMC6551394

[4] Gormsen J , Burcharth J, Gögenur I, et al. Prevalence and risk factors for chronic abdominal pain after roux-en-Y gastric bypass surgery: a cohort study. Ann Surg 2021;273:306–14. 10.1097/SLA.0000000000003356. 31058699

[5] Chahal-Kummen M , Våge V, Kristinsson JA, et al. Chronic abdominal pain and quality of life after roux-en-Y gastric bypass and sleeve gastrectomy – a cross-cohort analysis of two prospective longitudinal observational studies. Surg Obes Relat Dis 2023;19:819–29. 10.1016/j.soard.2023.01.020. 36870870

[6] Mala T . The gastric remnant in roux-en-Y gastric bypass: challenges and possibilities. J Clin Gastroenterol 2016;80:527–31. 10.1097/MCG.0000000000000550 27203428

[7] La Vella E , Hovorka Z, Yarbrough DE, et al. Bile reflux of the remnant stomach following roux-en-Y gastric bypass: an aetiology of chronic abdominal pain treated with remnant gastrectomy. Burger Obes Relat Dis 2017;13:1278–83. 10.1016/j.soard.2017.04.007. 28576682

[8] Ahmad BN , Ahmad WN, Aalam WK, et al. Duodenogastric reflux, an important cause of post cholecystectomy symptoms. JK Practitioner 2003;10:188–90.

[9] Am T , Morales F, Rovito P. Hepatobiliary scintigraphy as a diagnostic modality for gastroparesis of the bypassed stomach. Obes Surg 2007;17:414–5. 17546852 10.1007/s11695-007-9050-1

[10] Tog C , Liu DS, Lim HK, et al. Risk factors for delayed gastric emptying following laparoscopic repair of very large hiatus hernias. BJS Open 2017;1:75–83. 10.1002/bjs5.11. 29951609 PMC5989959

[11] Grant Black K , Cameron Maclellan W. The impact of gastroparesis on antireflux surgery. Foregut 2023;3:520–4.

